# Data on optical coherence tomography guidance for the management of angiographically intermediate left main bifurcation lesions

**DOI:** 10.1016/j.dib.2017.08.015

**Published:** 2017-08-30

**Authors:** Ilaria Dato, Francesco Burzotta, Carlo Trani, Andrea Romano, Lazzaro Paraggio, Cristina Aurigemma, Italo Porto, Antonio Maria Leone, Giampaolo Niccoli, Filippo Crea

**Affiliations:** Institute of Cardiology, Catholic University of Sacred Heart, Rome, Italy

**Keywords:** Frequency domain-optical coherence tomography, Intermediate left main bifurcation lesion, Percutaneous coronary intervention

## Abstract

The data presented in this article are related to the research article entitled “Optical coherence tomography guidance for the management of angiographically intermediate left main bifurcation lesions: early clinical experience” [1].

In this article we reports details about our clinical experience with frequency domain-optical coherence tomography (FD-OCT) guidance for the management of patients with left main (LM) bifurcation lesions of intermediate angiographic severity. LM patients were assessed by FD-OCT and, on the bases of the findings, managed by myocardial revascularization or conservative treatment (revascularization deferral). The observed outcomes support the feasibility of FD-OCT guidance for LM bifurcated lesions and call for further clinical evaluations in appropriately designed prospective studies.

**Specifications Table**

**Table:** 

**Subject area**	Cardiology
**More specific subject area**	Frequency domain optical coherence tomography analysis (FD-OCT) of left main bifurcation and percutaneous treatment
**Type of data**	Tables
**How data was acquired**	Data were acquired from a FD-OCT database of our Institution
**Data format**	Raw, Analyzed
**Experimental factors**	The two groups of treatment (revascularized and deferred) were compared according to FD-OCT features
**Experimental features**	Chi-square test and T-test
**Data source location**	Rome, Italy
**Data accessibility**	The data are available with this article
**Related research article**	This is a direct submission to Data in Brief

**Value of the data**

• The data present the FD-OCT analysis of LM bifurcation lesions performed dividing LM bifurcation area in three segments, that are distal LM, polygon of confluence (POC) and ostial left anterior descending artery (LAD) or left circumflex artery (LCX).
• A comparison between revascularized and deferred group according FD-OCT features is reported.
• Moreover, we reports data on principal features of percutaneous treatment of LM bifurcation.

## 1 Data

The dataset of this article provides principal FD-OCT features analyzed in the three segments of LM bifurcation. The Table 1 shows measured FD-OCT parameters of LM bifurcation according to LM bifurcation segment and treatment group and comparison statistical analysis.

**Table 1:** Comparison of quantitative FD-OCT analysis of left main bifurcation between revascularized and deferred groups.

**Variables**	**Revascularized group (n=64)**	**Deferred group (n=58)**	**P**
**LM (n=122)**			
Cap thickness (µm)	108±91	88±85	0.3
RLA (mm^2^)	13.3±4.9	15±5.6	0.3
MLA (mm^2^)	7.6±4.5	9.6±4.6	0.06
AS (%)	41±28	34±24	0.09
**POC (n=122)**			
Cap thickness (µm)	134 ±101	121±76	0.9
Longitudinal SB ostium length (mm)	2.3±0.9	2.6±1.1	0.2
**Ostial LAD (n=103)**			
Cap thickness (µm)	120±67	108±85	0.4
RLA (mm^2^)	8.0±3.7	8.6±2.9	0.5
MLA (mm^2^)	3.2±1.7	4.9±2.2	0.001
AS (%)	55±19	40±18	0.001
**Ostial LCX (n=19)**			
Cap thickness (µm)	112±26	160±91	0.5
RLA (mm^2^)	7.4±3.9	7±1	0.2
MLA (mm^2^)	3.2±1.9	3.3±0.9	0.6
AS (%)	58±10	50±15	0.1

LAD= left anterior descending artery; LCX= left circumflex artery; POC= polygon of confluence; LM= left main coronary artery; RLA= reference luminal area; MLA =minimal lumen area; AS= area stenosis; SB= side branch.

In Table 2 a complete description of percutaneous revascularization procedure is reported.

**Table 2:** Distal left main PCI features (48 patients).

**Variables**	**n (%)**
DES	48 (100)
Type of stent: Zotarolimus-eluting stent Everolimus-eluting stent Biolimus-eluting stent	40 (83) 6 (13) 2 (4)
Nr of stents: 1 2	38 (79) 10 (21)
Mean stent length (mm)	28±9
Mean stent diameter (mm)	3.7±0.5
Bifurcation stenting technique Provisional and inverted provisional T/ TAP stenting	43 (90) 5 (10)
Postdilation	47 (98)
Mean postdilation balloon diameter (mm)	4.4±0.5
Final kissing balloon	37 (77)
Ad hoc PCI	11 (23)

PCI=percutaneous coronary intervention; DES= drug eluting stenting; TAP= T And small Protrusion technique.

## 2 Experimental design, materials and methods

### 2.1 Optical coherence tomography acquisition technique and analysis

We retrospectively identified from the FD-OCT database of our Institution all patients who consecutively underwent FD-OCT assessment of de novo angiographically intermediate stenosis of LM bifurcation. FD-OCT images were acquired with a commercially available system (C7 System and C7 Dragonfly; LightLab Imaging Inc/St Jude Medical, Westford, MA, USA),

from one of the two principal branches of LM (LAD or LCX). FD-OCT analysis was performed dividing LM bifurcation area in three segments, which are distal LM, POC and ostial LAD/LCX, as reported in previous study [1]. FD-OCT analysis was performed according the last consensus document on OCT imaging [2].

### 2.2 PCI features

Patient's clinical, angiographic and procedural data were prospectively recorded on a dedicated catheterization laboratory database and LM PCI features were analyzed.

### 2.3 Statistical analysis

Continuous variables were reported as mean ± standard deviation and compared with analysis of variance (Student's t test). Categorical variables were expressed as frequencies and compared with χ2 test. Normality of data was determined using the D’Agostino-Pearsons test and verified using histogram plots. A two-sided P value of 0.05 was considered significant.

## Funding sources

This work is a part of a Ph.D. thesis of Ilaria Dato and was funded by St. Jude Medical (research grant 25520).
