# Supplementary material for: Data on optical coherence tomography guidance for the management of angiographically intermediate left main bifurcation lesions
Source: Data Brief. 2017 Aug 30;14:635–8. doi: 10.1016/j.dib.2017.08.015 (PMC5587878; doi:10.1016/j.dib.2017.08.015)
Supplement: Supplementary file 1 — Supplementary material [file mmc1.docx]

**AUTHOR DECLARATION**

**TITLE: DATA ON OPTICAL COHERENCE TOMOGRAPHY GUIDANCE FOR THE MANAGEMENT OF ANGIOGRAPHICALLY INTERMEDIATE LEFT MAIN BIFURCATION LESIONS**

**AUTHORS:** Ilaria Dato_1_ MD PhD, Francesco Burzotta_1_ MD PhD, Carlo Trani_1_ MD, Andrea Romano_1_ MD, Lazzaro Paraggio_1_ MD, Cristina Aurigemma_1_ MD PhD, Italo Porto_1_ MD PhD, Antonio Maria Leone_1_ MD PhD, Giampaolo Niccoli_1_ MD PhD, Filippo Crea_1_ MD PhD

^1^Institute of Cardiology, Catholic University of Sacred Heart, Rome, Italy

We wish to draw the attention of the Editor to the following facts which may be considered as potential conflicts of interest and to significant financial contributions to this work.

- Dr Dato discloses to have received in the past a research grant from St. Jude Medical. Dr Burzotta, Trani, Niccoli, Leone, Aurigemma and Porto disclose to have received speaker’s fees from St. Jude Medical.

- We confirm that the manuscript has been read and approved by all named authors and that there are no other persons who satisfied the criteria for authorship but are not listed. We further confirm that the order of authors listed in the manuscript has been approved by all of us.

- We confirm that we have given due consideration to the protection of intellectual property associated with this work and that there are no impediments to publication, including the timing of publication, with respect to intellectual property. In so doing we confirm that we have followed the regulations of our institutions concerning intellectual property.

- We understand that the Corresponding Author is the sole contact for the Editorial process (including Editorial Manager and direct communications with the office). He is responsible for communicating with the other authors about progress, submissions of revisions and final approval of proofs. We confirm that we have provided a current, correct email address which is accessible by the Corresponding Author and which has been configured to accept email from ([f.burzotta@rm.unicatt.it](mailto:f.burzotta@rm.unicatt.it)).
